# Supplementary figures and images for: Facilitating Drug Discovery in Breast Cancer by Virtually Screening Patients Using In Vitro Drug Response Modeling
Source: Cancers (Basel). 2021 Feb 20;13(4):885. doi: 10.3390/cancers13040885 (PMC7924213; doi:10.3390/cancers13040885)

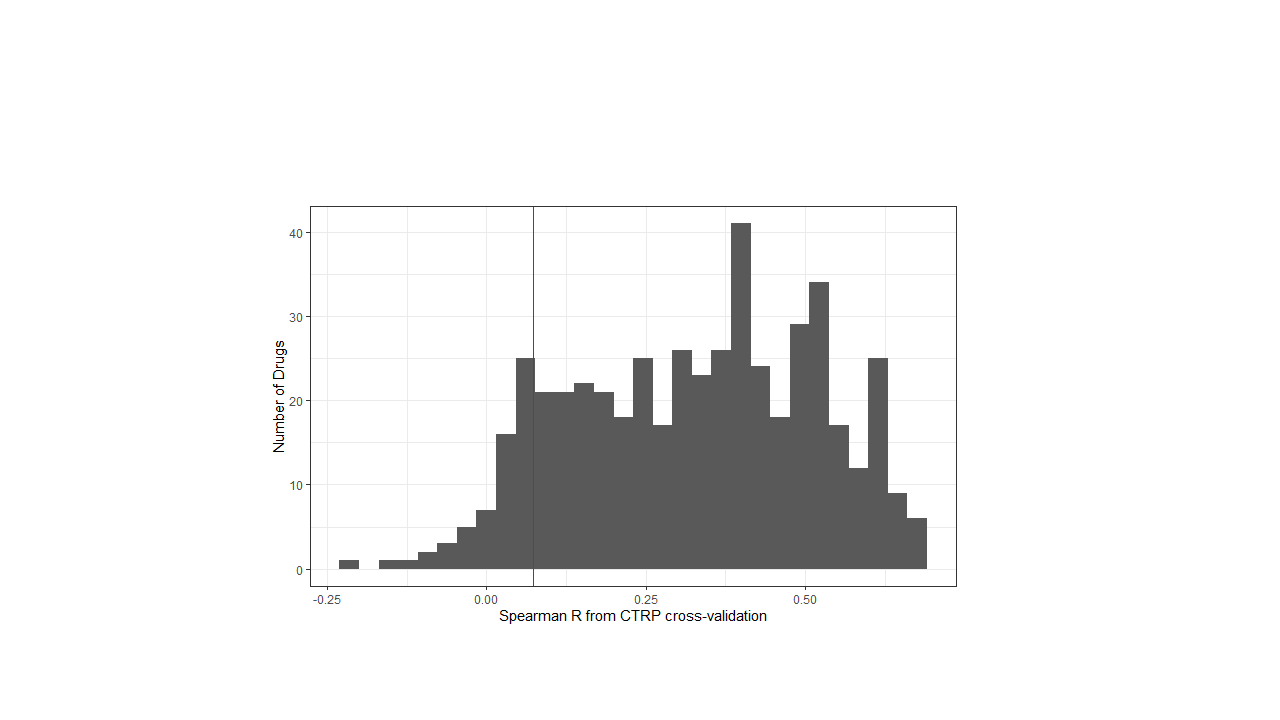

Supplement: Supplementary file 1 [file cancers-13-00885-s001.zip › Supplmenetal_Files/Supp Fig 1_ Distribution of Cross-validation results.TIF]

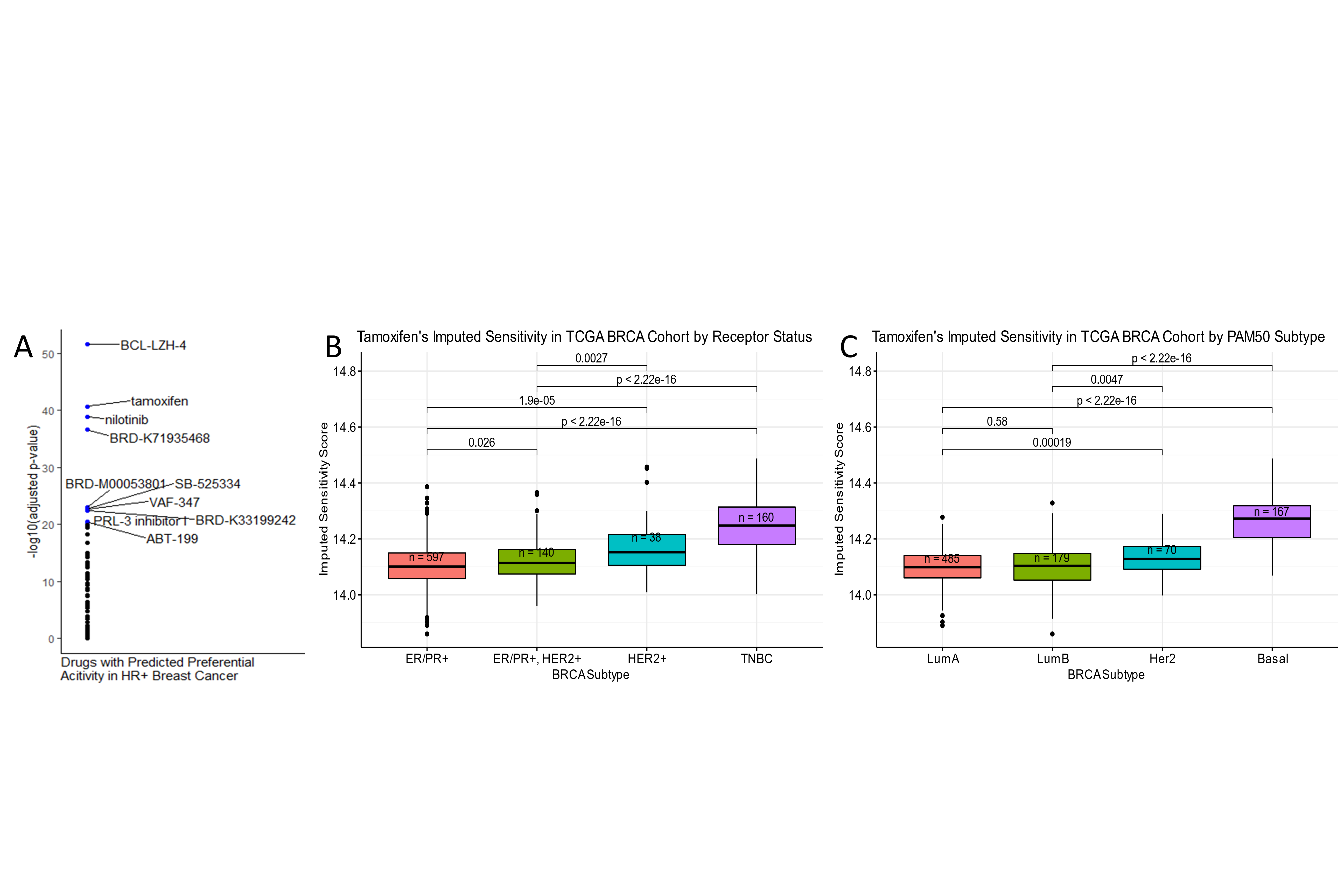

Supplement: Supplementary file 1 [file cancers-13-00885-s001.zip › Supplmenetal_Files/Supp Fig 2_Proof-of-concept Tamoxifen imputed efficacy against ER.TIF]

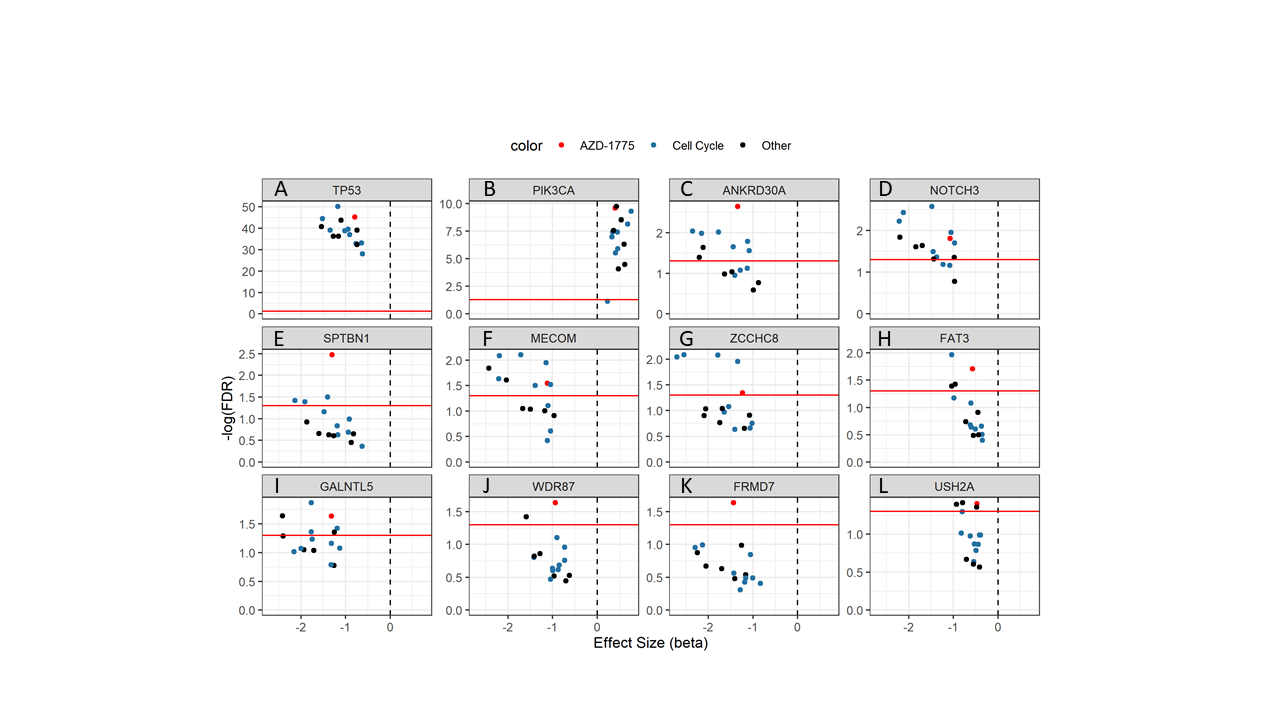

Supplement: Supplementary file 1 [file cancers-13-00885-s001.zip › Supplmenetal_Files/Supp Fig 3_Genomic associations of imputed response.TIF]

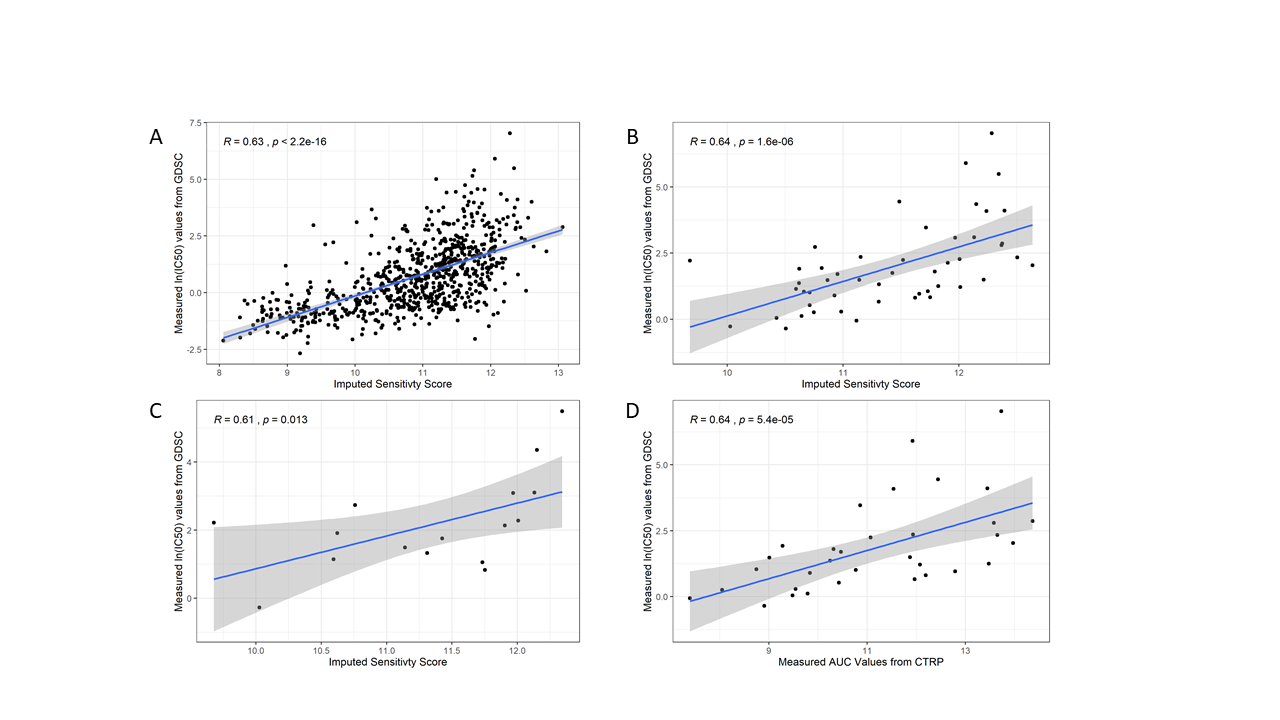

Supplement: Supplementary file 1 [file cancers-13-00885-s001.zip › Supplmenetal_Files/Supp Fig 4_Additional Correlations of imputed and measured response.TIF]

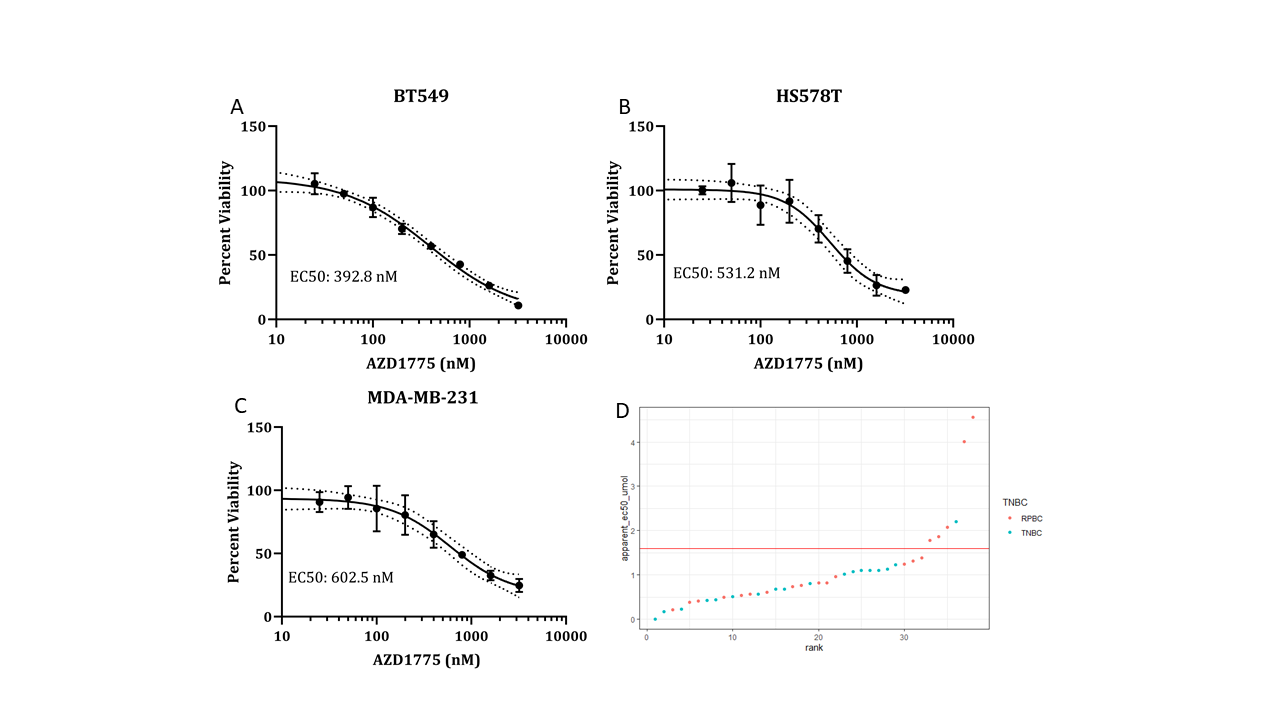

Supplement: Supplementary file 1 [file cancers-13-00885-s001.zip › Supplmenetal_Files/Supp Fig 5_AZD1775 in vitro.TIF]
